# A Systematic Review of Studies Eliciting Willingness-to-Pay per Quality-Adjusted Life Year: Does It Justify CE Threshold?

**DOI:** 10.1371/journal.pone.0122760

**Published:** 2015-04-09

**Authors:** Khachapon Nimdet, Nathorn Chaiyakunapruk, Kittaya Vichansavakul, Surachat Ngorsuraches

**Affiliations:** 1 Faculty of Pharmaceutical Sciences, Prince Songkla University, Hatyai, Thailand; 2 School of Pharmacy, Monash University Malaysia, Selangor, Malaysia; 3 Center of Pharmaceutical Outcomes Research (CPOR), Department of Pharmacy Practice, Faculty of Pharmaceutical Sciences, Naresuan University, Phitsanulok, Thailand; 4 School of Pharmacy, University of Wisconsin, Madison, United States of America; 5 School of Population Health, University of Queensland, Brisbane, Australia; Deakin University, AUSTRALIA

## Abstract

**Background:**

A number of studies have been conducted to estimate willingness to pay (WTP) per quality-adjusted life years (QALY) in patients or general population for various diseases. However, there has not been any systematic review summarizing the relationship between WTP per QALY and cost-effectiveness (CE) threshold based on World Health Organization (WHO) recommendation.

**Objective:**

To systematically review willingness-to-pay per quality-adjusted-life-year (WTP per QALY) literature, to compare WTP per QALY with Cost-effectiveness (CE) threshold recommended by WHO, and to determine potential influencing factors.

**Methods:**

We searched MEDLINE, EMBASE, Psyinfo, Cumulative Index to Nursing and Allied Health Literature (CINAHL), Center of Research Dissemination (CRD), and EconLit from inception through 15 July 2014. To be included, studies have to estimate WTP per QALY in health-related issues using stated preference method. Two investigators independently reviewed each abstract, completed full-text reviews, and extracted information for included studies. We compared WTP per QALY to GDP per capita, analyzed, and summarized potential influencing factors.

**Results:**

Out of 3,914 articles founded, 14 studies were included. Most studies (92.85%) used contingent valuation method, while only one study used discrete choice experiments. Sample size varied from 104 to 21,896 persons. The ratio between WTP per QALY and GDP per capita varied widely from 0.05 to 5.40, depending on scenario outcomes (e.g., whether it extended/saved life or improved quality of life), severity of hypothetical scenarios, duration of scenario, and source of funding. The average ratio of WTP per QALY and GDP per capita for extending life or saving life (2.03) was significantly higher than the average for improving quality of life (0.59) with the mean difference of 1.43 (95% CI, 1.81 to 1.06).

**Conclusion:**

This systematic review provides an overview summary of all studies estimating WTP per QALY studies. The variation of ratio of WTP per QALY and GDP per capita depended on several factors may prompt discussions on the CE threshold policy. Our research work provides a foundation for defining future direction of decision criteria for an evidence-informed decision making system.

## Introduction

It is widely known that using cost-effectiveness (CE) threshold as a cut-off for deciding whether an intervention is cost-effective is not uncommon [1]. Despite a controversy whether the threshold should be set, the CE threshold has been used implicitly or stated explicitly in various countries [2–6]. After a number of years of using health technology assessment (HTA) as part of decision makings, the CE thresholds in some countries, e.g. UK and Australia, become more apparent [5,7,8]. Several methods, such as expert opinion, human capital, WTP, and WHO recommendation, were used to estimate WTP per quality-adjusted life year (QALY) values [2,9–11]. However, how to derive appropriate cut-offs is still inconclusive.

CE threshold is defined as the maximum value of money per health outcome that a jurisdiction decides to pay for adopting a technology or an intervention [1]. Various jurisdictions refer to World Health Organization(WHO) recommendation for their CE thresholds, which were based on one to three times the gross domestic product (GDP) per capita per disability–adjusted life years (DALYs) as a cut-off [10,11]. However, in the practice, the CE threshold unit was usually cost per QALY and most of CE threshold studies were based on QALY [12–14]. WTP per QALY, which stems from the maximum amount ones would be willing to pay in order to gain an additional QALY, is another economic concept that has been used to justify CE thresholds [9,15–18]. A number of researchers have conducted WTP per QALY studies to understand how patients or general population valued one QALY gained in various diseases [9,18–27]. However, there has not been any evidence revealing linkage or comparison between WTP per QALY and CE threshold based on WHO recommendation in any country. Having these linkages or comparisons would not only reflect decision makers’ justification of technology or intervention adoption but also help all stakeholders, including the pharmaceutical industry, to have a better understanding of decision criteria. In addition, although several previous stated preference studies revealed that WTP per QALY values varied depending on how scenarios were specified, the elicitation instrument used, and other factors [6,14,20,21,24–29], there has not been a comprehensive summary of literatures on how these factors affect WTP per QALY values. The objectives of this study were therefore to systematically review WTP per QALY literatures and compare WTP per QALY with CE threshold recommended by WHO for each country. Also, potential factors influencing the ratios between WTP per QALY and CE threshold were examined.

## Methods

### Data sources and search strategy

Various databases including MEDLINE, EMBASE, Psyinfo, Center of Research Dissemination (CRD), Cumulative Index to Nursing and Allied Health Literature (CINAHL), and EconLit were systematically searched. They were searched from their inception until 15 July 2014. Medical Subject Headings (MeSH) and keywords used for the search included 1) (willingness to pay or contingent valuation or discrete choice experiment) AND (quality adjusted life year or QALY), OR 2) willingness to pay for (per) quality adjusted life year. There were no language restrictions.

### Study selection and Data extraction

Studies were included if they met the following criteria: 1) an original article eliciting WTP per QALY, 2) using stated preference method, and 3) estimating WTP per QALY in health-related issues. Two investigators (K.N. and K.V.) independently reviewed each abstract, completed full-text reviews, and extracted information from each study for inclusion in study analysis. Data extracted from each study were year of publication, year of study, country, number of country per study, characteristics of hypothetical scenarios, number of scenarios per study, sample size, sampling method, mode of administration, interviewer, WTP elicitation method (WEM), number of WEM per study, utility elicitation method (UEM), number of UEM per study, types of respondents, respondents’ income, and WTP per QALY values. Since many studies were conducted in a number of countries and/or scenarios, it was possible that more than one WTP per QALY value was obtained from each of them. [24]. During data extraction, the trimmed median of WTP per QALY value was preferred to median, and the trimmed mean was preferred to mean. Since cost-derived data are generally skewed, median is preferred. The trimmed analysis value was selected because the outliers were excluded from the analysis [30].

### Data analysis

A descriptive analysis was conducted. All values were converted to US dollar units ($) in the year of study based on exchange rates from World Bank [31]. The WTP per QALY value was compared to GPD per capita, which was obtained from World Bank [32] for the year and country of study. As a result, the ratio of WTP per QALY value compared to GDP per capita was calculated. A number of factors were hypothesized to have affected the relationship between WTP per QALY and GDP per capita [23–26]. They included outcomes, perspectives, severity of hypothetical scenarios, utility elicitation method, duration of scenario, type of respondent, type of country income, and funding sources [20,23–27]. These relationships were tested by independent sample t-tests or ANOVA. All analyses were performed using SPSS 18.0 for Windows, (Chicago, Ill).

## Results

### Study selection

**Fig 1:**
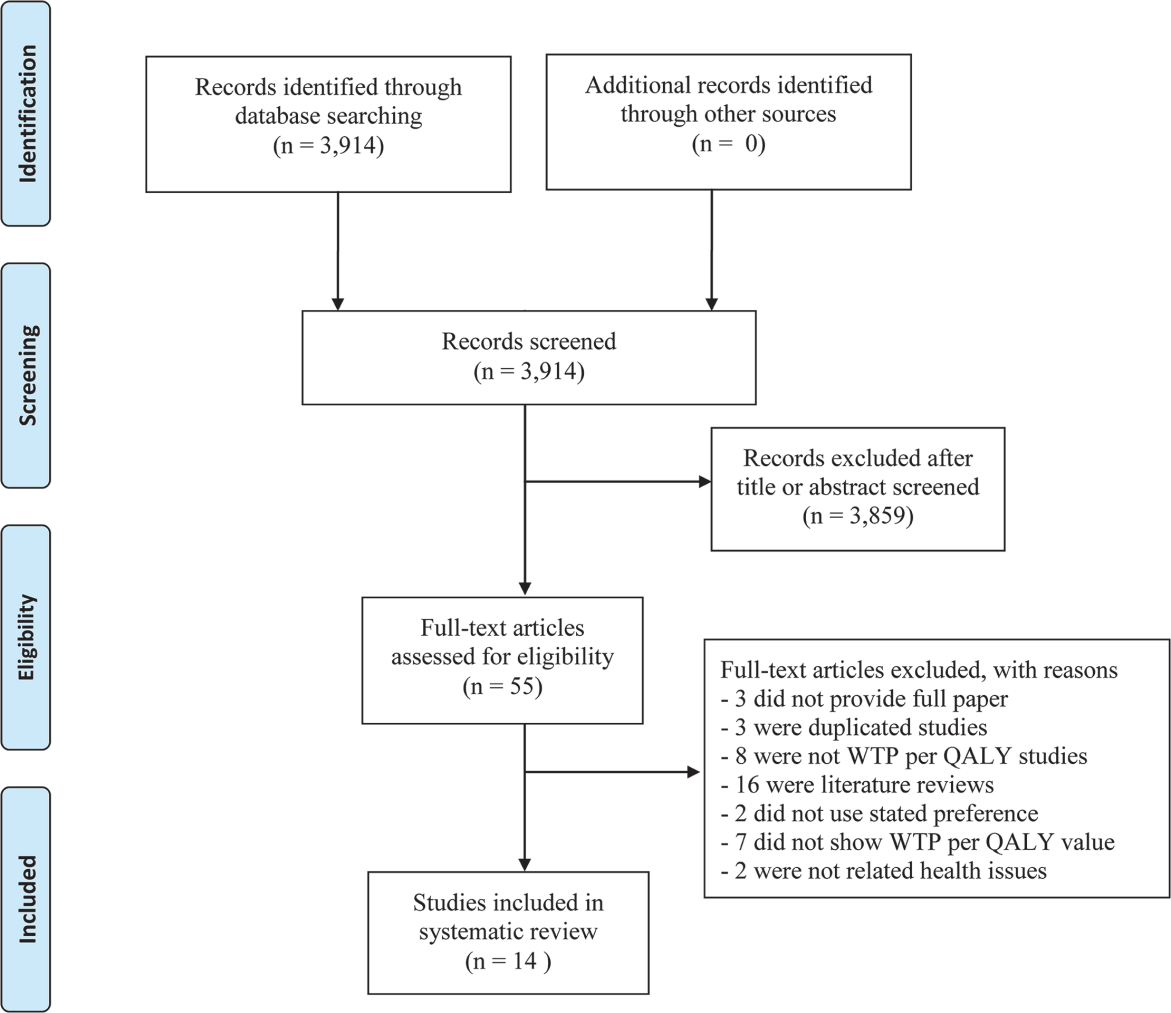
Flow diagram of study selection.

A total of 3,914 articles were identified (Fig 1). After exclusion of duplicated or irrelevant articles, 55 potentially relevant articles were retrieved for more detailed evaluation. We excluded 41 studies due to the following reasons: their full papers were not available (3), they were duplicated studies (3), they were not WTP per QALY studies (8), they were literature reviews (16), they did use stated preference to estimate WTP per QALY (2), they did not report WTP per QALY value (7), and they were not related to health issue (2). As a result, 14 articles were included into this review.

### Study Characteristics

Table 1 shows the study characteristics. The number of published WTP per QALY studies has grown rapidly over time; one study in 1995–1999, one study in 2000–2004, four studies in 2005–2009, and eight studies in 2010 to 2014. These studies were conducted in various geographical regions e.g. Europe (7/14, 50.00%) [19,20,22–24,29,33], Asia (3/14, 21.43%) [25–27], and United states (3/14, 21.43%) [21,28,34]. In addition, one study was (7.14%) conducted across four regions including Europe, America, Asia, and Australia [6]. Totally, WTP per QALY studies were conducted in 16 countries and all of them were cross-sectional studies. Only two of 16 countries (18.75%) were low and middle income countries including China and Thailand [26,27].

**Table 1:** Methodology difference in WTP per QALY.

**Study Characteristics**	**Frequency (%)**	**References**
Total number or articles reviewed	14	
**Publication year**		
** **1994–1999	1 (7.14)	[29]
** **2000–2005	3 (21.43)	[21,22,28]
** **2006–2010	4 (28.57)	[6,19,23,34]
** **2010–2014	6 (42.86)	[20,24–27,33]
**Region**		
** **Europe	7 (50.00)	[19,20,22–24,29,33]
** **Asia	3 (21.43)	[25–27]
** **America	3 (21.43)	[21,28,34]
** **Asia, Europe, America and Australia	1 (7.14)	[6]
**Sample respondent**		
** **General population	9 (64.29)	[6,19–26]
** **Patient	3 (21.43)	[28,29,33]
** **General population and Patient	2 (14.29)	[6,34]
**Sample size**		
** **101–500	3 (21.43)	[21,28,29]
** **501–1,000	4 (28.57)	[23,27,33,34]
** **More than 1,000	7 (50.00)	[6,19,20,22,24–26]
**Mode of administration**		
** **Interview	10 (71.43)	[19,20,22–24,26–29,33]
** **Internet	2 (14.29)	[6,25]
** **Internet and telephone	1 (7.14)	[34]
** **Interview and telephone	1 (7.14)	[21]
**Number of Scenarios per study**		
** **1 scenario	5 (35.71)	[6,27–29,33]
** **2–10 scenarios	3 (21.43)	[21,26,34]
** **11–20 scenarios	2 (14.29)	[23,25]
** **More than 20 scenarios	4 (28.57)	[19,20,22,24]
**WTP Elicitation Method (WEM)**		
** Contingent Valuation**		
** **Open-ended question	1 (7.14)	[21]
** **Bidding game	3 (21.43)	[27,28,33]
** **Card sorting procedure	2 (7.14)	[23,24]
** **Single-bounded dichotomous choice	1 (7.14)	[29]
** **Double-bounded dichotomous choice	2 (14.29)	[6,25]
** **Bidding game follow open-ended question	1 (7.14)	[34]
** **Payment scale follow open-ended question	1 (7.14)	[20]
** **Open-ended question or bidding game	1 (7.14)	[26]
** **Open-ended question and Payment card	1 (7.14)	[19]
**Discrete choice experiment**		
** **Closed-ended question	1 (7.14)	[22]
**Number of WEM per study**		
** **1 type	13 (92.86)	[6,20–29,33,34]
** **2 types	1 (7.14)	[19]
**Utility Elicitation Method (UEM)**		
**Not using UEM**	1 (7.14)	[6]
**Direct Method**		
** **Time-trade off (TTO)	1 (7.14)	[34]
** **Standard gamble (SG)	1 (7.14)	[23]
** **SG or TTO	1 (7.14)	[24]
** **TTO and VAS	1 (7.14)	[26]
** **Rating scale and TTO	1 (7.14)	[29]
** **TTO VAS and SG	2 (14.29)	[21,28]
**Indirect method**		
** **EQ-5D	3 (21.43)	[20,22,25]
** **EQ-5D and SF-36	1 (7.13)	[27]
**Direct method and Indirect method**		
** **Rescaling VAS and EQ-5D	1 (7.14)	[19]
** **VAS and EQ-5D	1 (7.14)	[33]
**Number of UEM per study**		
** **No	1 (7.14)	[6]
** **1 type	5 (35.71)	[20,22,23,25,34]
** **2 types	6 (42.89)	[19,24,26,27,29,33]
** **3 types	2 (14.29)	[21,28]

Nine studies (64.28%) [6,19–26] were conducted in a general population, while three studies (21.43%) [27–29] were in patients. However, respondents of two studies (14.29%) [27,34] were from both general and patient populations. Among 11 studies including general populations, subjects were randomly sampled in only six studies (54.50%) [6,21–23,25,26]. Three studies used stratified random sampling based on race [21], age, gender [25], region, and income level [26].

The number of respondents varied from 104 to 21,896 persons. The sample size was less than 500 in three studies (21.43%) [21,28,29] and more than 1,000 in seven studies (50.00%) [6,19,20,22,24–26]. The reported mean of age ranged from 32.20 to 65.40 years old. The percentage of females in most studies was 46.50–60.70%, while one study included only female patients [29]. Household income was reported in most studies (7/14, 50.00%) [19,20,25–27,29,33], while individual income were reported in only 14.28% of all studies [21,28]. The average individual incomes in all studies were less than the countries’ GDP per capita. Nevertheless, five studies (35.71%) did not report both average household and individual incomes.

### Health State Scenarios

Table 1 shows the differences of methods used in reviewed studies. The number of scenarios varied from one to 27 per study; however, approximately 35% of all studies (5/14) used only one scenario [6,27–29,33]. Out of 14 studies, only three studies (21.43%) [21,26,34] provided a full description of specific disease with severity, and disease duration as a hypothetical scenario. For example, Byne et al. clearly specified that patients had knee osteoarthritis (OA) until the end of life, with three levels of severity (mild OA, severe OA, and current health state) [21]. Lieu et al. used the scenarios of temporary herpes zoster with varying durations (one to 12 months) and pain intensity (pain scale of 0 to10) [34]. Thavorncharoensap et al. asked respondents to imagine being in hypothetical scenarios including paralysis, blindness, and allergy for five years with two levels of severity for each clinical condition [26].

The hypothetical scenarios varied across studies. They included viral infection [20], knee OA [21], chronic health state [22], herpes zoster [34], serious illness [6], paralysis [26], blindness [26], allergy [26], symptoms without hormone replacement therapy [29], prostatitis [27], and current health state of each respondent [21,27,28,33]. EQ-5D was used to describe varying severity levels of hypothetical scenarios in seven studies [19–25]. The duration of hypothetical scenarios varied from one month until death. The duration of hypothetical scenarios, specified in half of the included studies, was less than one year [6,19,20,24,27,33,34]. Three studies had several scenarios and specified each health state with varying duration [23,25,34].

Respondents were asked to state their willingness to pay for treatments that could either improve quality of life or extend life or save life. Most studies (12/14, 85.71%) asked respondents to value QALY in the perspective of improving quality of life [19–24,26–29,33,34], while only one study (7.14%) asked respondents for the value of extending life [6] and another study asked for all improving quality of life, extending life, and saving life [25]. In addition, only one study had respondents elicit WTP for disease prevention [26].

Most studies (12/14, 85.71%) asked respondents to elicit willingness to pay for themselves (individual perspective) [19,21–29,33,34]. Only one study estimated the value of WTP per QALY for the others in society (societal perspective) [20], while another study included both individual and societal perspectives and compared their values [6].

### Mode of administration

The most common mode of administration was face-to-face interview (10/14, 71.43%) [19,20,22–24,26–29,33], followed by internet survey (2/14, 14.29%) [6,25], internet or telephone survey (1/14, 7.14%) [34], and face-to-face or telephone interview (1/14, 7.14%) [21]. Some studies reported that interviewers were trained nurses [29] or trained researcher assistants [21,28,33].

### Willingness to pay (WTP) elicitation methods

Almost all studies (13/14, 92.85%) used the contingent valuation method, and only one study used discrete choice experiments to estimate WTP [22]. Among the studies using the contingent valuation method, most studies (9/13, 69.23%) used only one elicitation method to estimate WTP. They included open-ended question [21], a bidding game [27,28,33], a card sorting procedure [23,24], single-bounded dichotomous choice [29], and double-bounded dichotomous choice [6,25]. On the other hand, the rest (4/13, 30.77%) used two elicitation methods [19,20,26,34]. For instance, Bobinac et al. used both open–ended question and payment card. Lieu et al. combined a biding game and open-ended question [34], while Bobinac et al. combined a payment scale and open-ended question to elicit WTP values [20]. In another study, Thavorncharoensap et al. used a bidding game method and open-ended questions when the willingness to pay was less than the minimum offered bid or higher than the maximum offered bid [26]. Almost all studies (7/8, 87.50%) [6,25–29,33,34] using either bidding game or dichotomous choice used randomization for the first bid value [6,25–27,29,33,34] to minimize starting point bias.

### Utility elicitation methods

Time trade off (TTO) and standard gamble (SG) were used in six studies [21,24,26,28,29,34], and four studies [21,23,24,28], respectively, while EQ-5D were used in six studies [19,20,22,25,27,33] and only one study [29] used SF-36. Six studies (42.85%) used two utility elicitation methods to estimate the utility of health state in scenario [19,24,26,27,29,33]. Only two studies (14.29%) used both direct and indirect preference based measures [19,33]. For instance, Bobinac et al. used rescaling VAS and EQ-5D [19], and Martin-Fernandez et al. used VAS and EQ-5D [33]. While five studies (35.71%) used only one utility elicitation method [20,22,23,25,34], two studies (14.29%) used three methods including TTO, VAS, and SG [21,28]. However, Shiroiwa et al. did not use any utility elicitation method because their scenarios were composed of only death state (utility = 0) and perfect health state (utility = 1) [6].

### Comparisons between WTP per QALY and GDP per capita

All WTP per QALY values and their comparisons with GDP per capita of each study’s country are shown in Table 2. A total of 167 WTP per QALY values were obtained from the 14 studies. Based on varying scenarios, utility and WTP elicitation methods, and perspectives, these values varied extensively. Overall, these studies reported that WTP per QALY values fell between $2,019 [21] and $282,821 [20]. The mean (SD) and median of WTP per QALY values were $34,309 ($55,390), $9921, respectively. When WTP values were compared to GDP per capita of each country for specific study years, the ratios of WTP per QALY and the country’s GDP per capita ranged from 0.05 [21] to 5.40 [20]. The mean (SD) and median values of these ratios were 0.77 (0.89) and 0.43, respectively. Interestingly, among 167 observed values of WTP per QALY, more than three quarters of the number of these values (127/147, 86.39%) were below one GDP per capita for an additional QALY.

**Table 2:** WTP per QALY compared to GDP per Capita.

**Study**	**Year of Study**	**Hypothetical Scenario**	**Outcome**	**No of Scenarios**	**No of WTP/QALY**	**Country**	**Respondent**	No of Respondents	WTP/QALY ($)	WTP/QALY compared to GDP per capita (times)
**Contingent valuation**										
** **Zethraeus[29]	1995	HRT	Improving QoL	1	2	Sweden	Patient	104	120,000–160,000	0.63–0.84
** **Byrne et al[21]	2001	Knee osteoarthritis¶	Improving QoL	3	9	US	GP	193	2,019–35,257	0.05–0.95
		Current HS ^^^^								
** **Lieu et al[34]	2005	Herpes zoster	Improving QoL	9	9	US	Patient	474	26,000–45,000	0.52–1.02
							GP	478		
** **Pinto-Prade et al[23]	2007	EQ-5D HS	Improving QoL	13	13	Spain	GP	560	11,999–182,134	0.26–3.91
** **Thavornchareonsap et al[26]	2008	Allergy, Paralysis, and Blindness	Improving QoL	12	12	Thailand	GP	1,191	1,698–17,283	0.20–2.06
** **Bobinac et al[19]	2008	EQ-5D HS	Improving QoL	29	4	Netherland	GP	1,091	13,360–34,097	0.25–0.64
** **Shiroiwa et al[6]	2009	Serious illness	Extending life	4	3	Japan	GP	1,114	44,000–47,000	1.16–1.23
		Serious illness	Extending life	4	3	ROK	GP	1,000	79,000–90,000	3.42–3.90
		Serious illness	Extending life	4	3	Taiwan	GP	504	70,000–84,000	4.03–4.82
		Serious illness	Extending life	4	3	UK	GP	1,002	39,000–61,000	0.81–1.28
		Serious illness	Extending life	4	3	Australia	GP	1,000	50,000–68,000	1.22–1.66
		Serious illness	Extending life	4	3	US	GP	1,000	56,000–66,000	1.16–1.36
** **Shiroiwa et al [25]	2009	EQ-5D HS	Improving QoL	12	12	Japan	GP	2,400	24,375–121,876	0.53–2.64
		Dead state	Extending life	2	2	Japan	GP	2,400	70,567–71,913	1.52–1.56
		Dead state	Saving life	2	2	Japan	GP	2,400	37,797–72,317	0.82–1.56
** **Zhao et al [27]	2009	Current HS€	Improving QoL	1	2	China	GP	364	4,711–5,012	0.72–0.77
		Current HS	Imoroving QoL	1	2	China	Patient	286	7,408–7,306	1.12–1.13
** **Bobinac et al[20]	2010	Viral illness	Improving QoL	29	2	Netherland	GP	1,004	159,810–252,408	3.42–5.40
** **King et al[28]		Current HS^^^	Improving QoL	1	3	US	Patient	391	12,500–32,200	0.32–0.81
** **Robinson et [24]	2010	EQ-5D HS	Improving QoL	27	8	Netherland	GP	2,510	3,412–7,904	0.07–0.17
		EQ-5D HS	Improving QoL	27	8	UK	GP	2,312	6,775–3,256	0.09–0.18
		EQ-5D HS	Improving QoL	27	8	France	GP	2,674	3,256–6,775	0.08–0.18
		EQ-5D HS	Improving QoL	27	8	Spain	GP	2,697	6,671–12,669	0.22–0.43
		EQ-5D HS	Improving QoL	27	8	Sweden	GP	2,604	3,235–7,842	0.07–0.16
		EQ-5D HS	Improving QoL	27	8	Norway	GP	2,020	7,659–15,472	0.09–0.18
		EQ-5D HS	Improving QoL	27	8	Denmark	GP	2,637	5,749–15,409	0.10–0.27
		EQ-5D HS	Improving QoL	27	8	Poland	GP	2,173	3,611–10,744	0.29–0.87
		EQ-5D HS	Improving QoL	27	8	Hungary	GP	2,287	3,611–10,748	0.24–0.60
** **Martin-Fernandez[33]	2011	Current HS	Improving QoL	1	2	US	Patient	757	10,119–10,305	0.42–0.43
**Discrete choice experiment**										
** **Gryd-Hasen[22]	2001	Chronic HS*	Improving QoL	23	1	Denmark	GP	3,201	10,972	0.37

Abbreviations: GDP, gross domestic product; No, number; HRT, hormone replacement therapy; HS, health state, No, number; GP, general population; QoL, quality of life;UK, united Kingdom;

ROK, Republic of Korea; US, United states;

¶ mild or severe osteoarthritis;

*EQ-5D described severity;

^^^ CSM,general medical clinic,cerebral aneurysms;

^^^^Knee osteoarthritis;

€chronic prostatitis

### Associations between factors and the ratios of WTP per QALY and GDP per capita

Table 3 shows the results of the relationship between various factors and the ratio of WTP per QALY compared to GDP per capita. The average ratio of WTP per QALY and GDP per capita for extending life or saving life (2.03) was significantly higher than the average for improving quality of life (0.59) with the mean difference of 1.43 (95% CI, 1.81 to 1.06). It was also found that, on average, the estimates from a societal perspective (2.16) were clearly higher than those from an individual perspective (0.63) (p-value <0.01). A linear trend of the ratios of WTP per QALY and GDP per capita was proportional to the increasing severity of conditions (p-value <0.01). WTP per QALY and GDP per capita derived from indirect utility elicitation method (1.45) was significantly higher than direct utility elicitation method (0.45) (p-value <0.01). The ratio of WTP per QALY and GDP per capita from studies in LMIC (0.97) was insignificantly higher than that in non-LMIC (0.75) (p-value = 0.35).

**Table 3:** Exploration of the relationship between numerous factors associated with the ratio of WTP per QALY compared to GDP per capita.

Factors	Mean of WTP per QALY compared to GDP per capita **+** SD (times)	Mean difference (times) (95% CI)	P value
**Scenario characteristic**			
** Outcomes of all counties**			
** **Improving quality of life (n = 145)	0.59 + 0.74	§	
** **Extending life and Saving Life (n = 22)	2.03 + 0.98	-1.43 (-1.81 to -1.06)	< 0.01
** Perspective**			
** **Individual perspective (n = 153)	0.63 + 0.73	§	
** **Societal perspective (n = 12)	2.16 + 1.41	-1.53 (-2.00 to -1.06)	< 0.01
** Severity of hypothetical scenario**			
** **Mild severity (utility > 0.70) (n = 63)	0.37 + 0.35	§	
** **Moderate severity (utility 0.35–0.70) (n = 68)	0.70 + 0.91	-0.32 (-0.61 to -0.03)	0.03
** **High Severity (utility < 0.35) (n = 36)	1.65 + 1.22	-1.28 (-1.62 to -0.93)	< 0.01
** Utility elicitation method**			
** **Direct method (n = 124)	0.45 + 0.53	§	
** **Indirect method (n = 25)	1.45 + 1.05	-1.00 (-1.28 to -0.72)	< 0.01
** Duration of scenario**			
** **1 month to 1 year (n = 20)	1.30 + 0.58	§	
** **More than 1 year (n = 147)	0.71 + 0.98	0.59 (0.15 to 1.02)	0.01
**Respondent characteristic**			
** Type of respondent**			
** **Public (n = 140)	0.82 + 1.03	§	
** **Patient (n = 9)	0.70 + 0.30	0.12 (-0.53 to 0.77)	0.72*
** **Public and Patient (n = 18)	0.51 + 0.40	0.31 (-0.17 to 0.78)	0.96*
**Other factors**			
** Type of country income**			
** **Low and middle income countries (n = 16)	0.97 + 0.51	§	
** **Non-low and middle income countries (n = 151)	0.75 + 0.92	0.35 (-0.24 to 0.68)	0.35*
** Funding**			
** **Non drug company support (n = 42)	0.80 + 0.73	§	
** **Drug company support (n = 36)	1.62 + 1.39	-0.82 (-1.31 to -0.33)	0.01
** Sample size**			
** **Increment of 1,000 subjects		-0.30 (-0.43 to -0.17)	< 0.01

Abbreviations: GDP, gross domestic product; SD, standard deviation; CI, confidence interval; n, number of study

* no statistical significance

§ Comparator

Duration of scenario was significantly associated with the ratio between WTP per QALY and GDP per capita (p-value<0.01). The shorter duration (1 month to 1 year) scenario seemed to have higher WTP per QALY (p-value<0.01). Interestingly, the studies funded by drug companies reported that the ratio of WTP per QALY and GPD per capita (1.62) was higher than the ratio (0.80) in the other studies that were not funded by any drug company (p-value< 0.01). We also found that the sample size has a statistically significant negative association with the ratio of WTP per QALY and GPD per capita (0.30 per increment of 1,000 subjects).

## Discussions

To our best of knowledge, this is the first study systematically reviewing literatures on WTP per QALY that determined whether evidences justified the CE threshold recommended by WHO. The review provided the summary of methods that could be used for future improvement in this kind of study. In addition, it shed light on the relationship between WTP per QALY and GDP per capita. The comparison between results from research on WTP per QALY and CE threshold would be valuable to policy makers because they could use this evidence to support direction of future decisions. Even though this study did not reveal how WTP per QALY differed from the current thresholds used in their jurisdictions, policy makers could refer these numbers with their ‘implicit’ thresholds eventually.

There has been an increasing trend in the number of studies for WTP per QALY in the last decade. However, only 14 studies were included. The main reasons were that several studies were literature reviews [9,15,35–39] or did not report WTP per QALY values [40–46] or were not related to health issues [47,48]. Half of reviewed studies were conducted in European countries. A reason could be that many countries there adopted HTA for decision making and they have had a strong network, e.g. EUnetHTA, to conduct this type of study. In terms of methods used in these studies, this review shed light on various parts of study design, including samples, method of administration, scenarios, etc. For study samples, most studies used general population. Those researchers might perceive that health care as a public goods and believed that focusing on individual patients or diseases would not reflect the complete picture of society. This is not meant to say that WTP per QALY for particular diseases were not useful since in fact it could be used for other purposes. For instance, it could be used for bridging cost-effectiveness/cost-utility analysis with cost-benefit analysis, which has stronger theoretical ground, in particular diseases [16,49]. In other words, selected samples should depend on study objectives or applications. However, when these WTP per QALY values were compared with GDP per capita, their ratios were not significantly different. Certainly, it could not be generalized but it provided less concern for future use of research results from different types of samples. The results showed that these samples were asked to use either individual or societal perspectives when they responded to the questions. Most studies asked them to use their own perspectives since it might be easier for them to imagine from given scenarios and their responses should be more valid. The ratio between WTP per QALY and GDP per capita from two different perspectives were significantly different. This systematic review did not intend to determine which perspective would be better than another, but it suggested that perspective used in the study affected WTP per QALY values.

Our findings provided scientific evidences for the controversy of the use of a fixed CE threshold versus flexible CE thresholds [7,18,25]. For example, US used a fixed CE threshold at $US 50,000 [2], while the Netherlands applied different CE thresholds for interventions that aimed for life threatening conditions and for other conditions [50,51]. The ratios between WTP per QALY and GDP per capita varied substantially especially those for extending life or saving life and improving quality of life and the ratios were higher among those scenarios with severe conditions compared to mild conditions. These implied that perhaps a fixed CE threshold might not be appropriate or one CE threshold might not fit all circumstances. In addition, the results showed that the ratios between WTP per QALY and GDP per capita were 0.59 and 2.03 for improving quality of life and extending life or saving life, respectively. It is also important to note that all evidences on this difference were driven by studies conducted in non-LMIC since there was no study conducted in LMIC to determine WTP per QALY for extending life or saving life and improving quality of life. This is suggestive of the need for such a study to look at this aspect in LMIC. Currently, the interest in using different CE thresholds has been adopted in some countries [50,51].

The review showed the WTP elicitation method primarily was contingent valuation (CV). However, CV itself was composed of several types of methods. Among them, the bidding game was used slightly more frequently than others. One reason could be that the bidding game was similar to or based on the concept of standard gamble, which has been well known among health economists. However, CV methods have been criticized for various weaknesses [52]. For instance, respondents were asked to consider whole health states or scenarios in CV and decide how much they would like to pay. In reality, they might consider these health states or scenarios based on only some important attributes that were important to them. Among the reviewed studies, only one selected study used discrete choice experiments (DCE). A potential reason was that DCE might have just been introduced to the field of health economics [53–55]. However, DCE is based on a rigorous theory, random utility theory, which has recently been proven to measure utility well. Potentially, DCE could be used more for future research of WTP per QALY.

Direct methods, e.g. SG, TTO, VAS, or their combinations, were used more frequently. Only four studies used indirect methods, e.g. EQ-5D and SF-36. There were at least two possible explanations. First, using indirect method required scale tariffs, which might not be available in those countries. In addition, when these indirect methods were used, they needed to be validated with study samples. Therefore, using an indirect method in this case might not be efficient or convenient, as an exchange for the validity of utility measurement. On the other hand, using direct methods would not only provide studies strong theoretical ground but also allow the study results to relate to other studies’ results.

Another interesting result, that could stimulate controversy, was that the ratios between WTP per QALY and GDP per capita from different types of funding were significantly different. Those studies funded by drug companies tended to have higher ratio. This was by no means intended to reflect bias. Instead, it should be noted that the source of funding could have an impact on WTP per QALY, as compared to GDP per capita. In addition, the significant negative association of sample size and WTP per QALY compared to GDP per capita were worth noting. The higher ratios between WTP per QALY and GDP per capita in the small sample size might be due to the small study effect [56], which is a phenomenon of higher value of results among studies with smaller sample size. Subjects included in small studies might be selected in a way that is prone to give the higher value results.

There were several factors influencing with WTP per QALY values including severity of hypothetical scenario, outcomes, and duration of scenario. Therefore, we recommended these factors should be presented and clearly explained to respondents in future stated preference studies.

This review included only stated preference studies. Stated preference method is useful since it can derive WTP per QALY values for a number of specific scenarios. However, subjects may face difficulty imagining for scenarios. It could be challenging to have imagination without a full description of all relevant components of scenarios including hypothetical scenarios, severity, scenario outcomes, and duration. However, having specified a wide range of scenario, studies using stated preferences method could be derived to inform decision making on varying conditions in scenarios. This is contradictory to the use of the revealed preference method in which WTP per QALY can be derived from certain conditions or situations, providing fewer insights for supporting informed decision making.

Some researchers argued that the WTP per QALY estimated from stated preference method might not be relevant for policy making [16,49,57]. However, another researcher argued that countries needed to use a robust and simple method to look for WTP threshold for a QALY since it could be used to inform the political debate for the allocation of health care resources [6,18–28,33,34,37,58,59]. A number of countries have estimated WTP per QALY using stated preference for support policy decision making [6,24].

Finally, there has been a debate on the usefulness and limitation of the use of WTP per QALY for policy decision making [9,49,57,59]. An important issue is the elicitation of preference based on whose perspective. There were two studies estimating WTP per QALY values from societal perspective [6,20], while most studies focused on individual’s perspective. The issue of equity arose further among studies using individual perspective as the WTP values might be affected by individuals’ income. Therefore, WTP per QALY values derived from individuals might cause distributional issue problem especially for low income person or unemployed person. Even though the elicitation of preference from individuals was consistent with welfare economic theory, the individual’s valuation based on social perspective could provide information relevant for decision making under healthcare system [6,20,49,60].

A number of limitations should be acknowledged in this study. First, this study used the WHO recommendation as a reference CE threshold of each country since it was not feasible to identify their explicit CE. However, the WHO recommendation is recognized as the best available benchmark. Second, some studies used a number of scenarios and provided more than one WTP per QALY value, the average value might be weighted towards values from such studies more than those studies providing only one WTP per QALY value. Third, interaction effect of all factors was not controlled because of small sample size. It is noteworthy that mean difference may change if they had interaction.

## Conclusions

This systematic review provides a summary of all studies estimating WTP per QALY in the existing literature. A description of the similarities and differences on how studies have been conducted provides a good foundation for defining good practice for this kind of study. The variation of ratio of WTP per QALY and GDP per capita depended on several factors may prompt discussions on the CE threshold policy. Our findings provides pivotal evidence to enable policy makers to discuss and initiate conversations among themselves and stakeholders on how decisions can be made and what criteria decisions should be based on, so that the improved overall population health can be achieved through an evidence-informed health care decision making system.

## Supporting Information

S1 PRISMA Checklist PRISMA Checklist. (DOC)
